# Case Report: Two Cases of Salivary Duct Carcinoma in Workers With a History of Chromate Exposure

**DOI:** 10.3389/fmed.2021.730403

**Published:** 2021-10-18

**Authors:** Imran Seçin, Maike J. M. Uijen, Chantal M. L. Driessen, Carla M. L. van Herpen, Paul T. J. Scheepers

**Affiliations:** ^1^Department of Medical Oncology, Radboud Institute for Health Sciences, Radboud University Medical Center, Nijmegen, Netherlands; ^2^Department for Health Evidence, Radboud Institute for Health Sciences, Radboud University Medical Center, Nijmegen, Netherlands

**Keywords:** salivary duct carcinoma, salivary gland cancer, occupation, hexavalent chromium, paint

## Abstract

**Background:** Salivary duct carcinoma (SDC), one subtype of the 22 different salivary gland cancers, is a rare malignancy. Risk factors for the development of salivary gland cancer and SDC are largely unknown, although pollution has been described as one of the risk factors. In other cancers, especially in lung cancer, the carcinogenicity of chromium VI [Cr(VI)] is well-known. Here we report on two SDC patients who were occupationally exposed to Cr(VI) and discuss a potential relation between their Cr(VI) exposure and the occurrence of SDC.

**Case Presentation:** The work history of two SDC patients was analyzed for chemical exposures. Both patients had a history of Cr(VI) exposure, with maintenance of military equipment considered as the source for this exposure. Inhalation of Cr(VI) containing particles from the removal of old paint by mechanical abrasion was identified as a probable source of exposure for both patients, and one of these patients also applied new paint. Both patients reported not to have used any respiratory protection which may have resulted in substantial inhalation of Cr(VI)-containing chromates. Furthermore, in one patient inhalation of fumes from soldering may have resulted in relevant co-exposure.

**Conclusion:** A causal relation between Cr(VI) exposure and SDC, a rare cancer, cannot be demonstrated on an individual basis but detection in a population-based study is also unlikely because of the extremely low prevalence. Nevertheless, the work history is considered a relevant risk factor in the onset of SDC as occupational exposures to Cr(VI) occurred in poorly ventilated working environment and without using appropriate respiratory protective equipment.

## Introduction

Salivary duct carcinoma (SDC) is one of the most aggressive subtypes of salivary gland cancer. Salivary gland cancer is a rare cancer with an incidence rate of probably one to two adults in 100.000 each year (1), which makes SDC extremely rare, since <10% of all salivary gland cancer cases comprise of SDC (1–4). Due to its rarity, no studies investigated risk factors specifically for SDC. Some occupational exposures like radioactive substances and nickel compounds/alloys have previously been suggested as potential risk factors for salivary gland cancer (5). Salivary gland cancers are not considered hereditary, although an association between breast cancer and salivary gland cancer has been reported (6).

SDC is classified as an aggressive adenocarcinoma. Common genetic alterations in SDC include the *TP53* gene (53–68%), *PIK3CA* gene (18–26%), and *HRAS* gene (16%) (7). The vast majority (78–96%) of SDC tumors are positive for the androgen receptor (8–10). Furthermore, overexpression of human epidermal growth factor receptor 2 (*HER2neu*), is found in SDC in about 29–46% of the patients (10–12).

Hexavalent chromium [Cr(VI)] has been identified as a carcinogenic risk factor in humans and is known to increase the risk of lung cancer (13). Workers can become exposed to different chemical species of Cr(VI): chromic acid in chrome plating and the use of chromates in metal coatings, cement and in welding fumes. Due to their high chemical reactivity, direct skin contact with chromic acid and Cr(VI) containing chromates leads to irritation and may induce a skin allergy (14). Prolonged inhalation may lead to nasal irritation, hypertrophy of the nasal turbinates, and can even lead to ulceration and perforation of the nasal septum of chrome platers (15). Most other signs of toxicity are related to different diseases of the respiratory system including lung cancer. When Cr(VI) is inhaled, it can cross cell membranes of the lung and persist in the bifurcation of the lung for years after inhalation exposure. Due to its instability in the human body, Cr(VI) is reduced to the more stable Cr(III) by ascorbate, glutathione and other substances. During this process free radicals can be generated, which potentially causes structural DNA damage. Accumulation of scarcely water soluble Cr(VI)-containing particles in the bronchial bifurcation causes a sustained high tissue dose, which is believed to increase Cr(VI)-toxicity. The resulting increased cell proliferation may increase risk of tumorigenesis (15). Cancers of other sites are less frequent and more difficult to study because of the extremely low prevalence among chromium workers (13).

In this report we present two SDC patients and discuss a potential association with work-related Cr(VI) exposures.

## Case Descriptions

### Case 1

**Figure 1:**
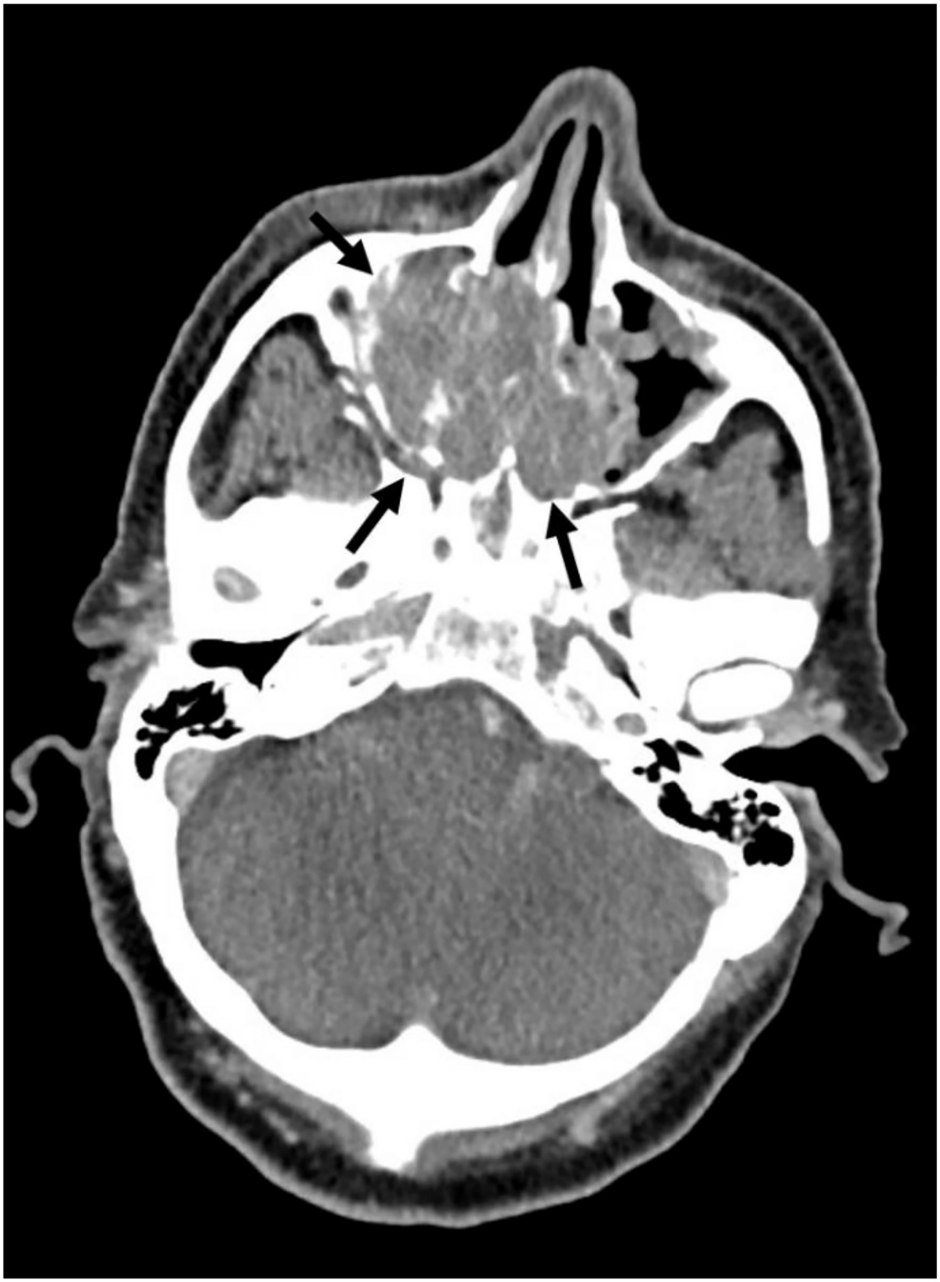
CT image of primary sinonasal SDC tumor (indicated by black arrows).

**Figure 2:**
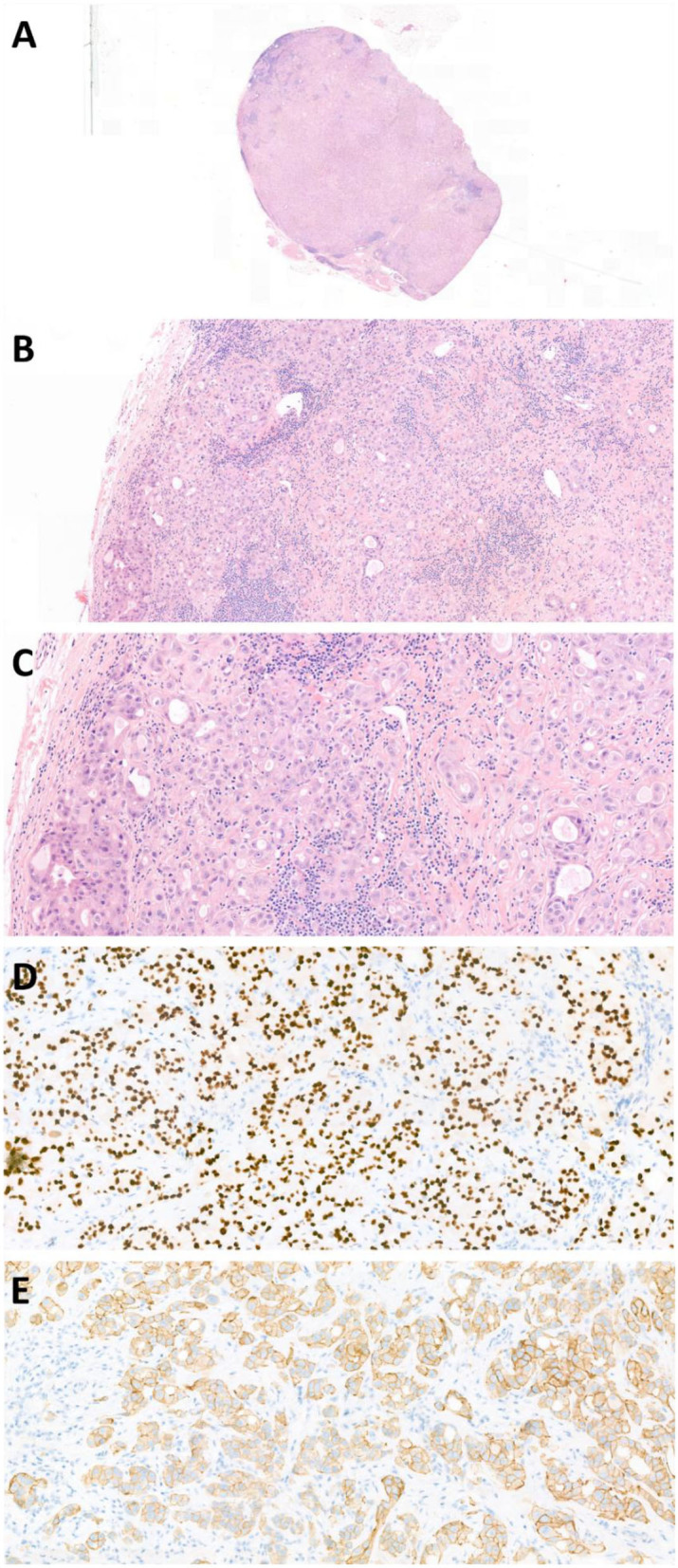
Photomicrographs of case report 1: cervical lymph node containing an SDC metastasis. **(A)** H&E stain 1X, **(B)** H&E stain 10X, **(C)** H&E stain 21X, **(D)** AR stain 20X, AR441 (BioCare Medical), **(E)** HER2neu stain 21X, HercepTest (Dako Agilent).

A 59-year-old male presented to the general practitioner because of painful ribs. Imaging, performed to rule out pulmonary embolism at the Emergency Department, showed no cause for his symptoms, but accidentally revealed enlarged lymph nodes in the neck. Histopathology obtained from a lymph node biopsy indicated a metastasis of an poorly differentiated adenocarcinoma. The node was almost entirely made up of an epithelial tumor which formed tubular structures. The tumor cells had ample eosinophilic cytoplasm and enlarged polymorphic anisochromatic and elucidated nuclei often with a prominent eosinophilic nucleolus. Both mitoses and apoptosis were observed. At the tumor border vascular invasion was present. Additional imaging examinations revealed a large sinonasal process (Figure 1). Subsequently, the patient was diagnosed with a primary SDC originating from the sinonasal cavity based on additional clinical information, histomorphology, and immunohistochemistry. Due to cervical and mediastinal lymph node metastases (T3N2bM0 disease), the patient was referred to our hospital, a tertiary referral center for salivary gland cancer, to discuss systemic treatment options. The tumor cells were positive for androgen receptor (AR) (see Figure 2). Human epidermal growth factor receptor 2 (*HER2neu*) status was assessed in accordance with the American Society of Clinical Oncology/College of American Pathologists (ASCO/CAP) guidelines for the evaluation of breast cancer (16). Immunohistochemistry (IHC) was inconclusive; IHC score: 2+, therefore fluorescence *in situ* hybridization (FISH) was performed which indicated no amplification, resulting in a negative HER2neu status. Additional molecular analysis was performed on a lymph node metastasis to detect other potential actionable targets for future systemic treatment options. The assay used for molecular analysis was the TruSight Oncology 500 (Illumina), which is a next-generation sequencing assay that enables comprehensive genomic profiling of 532 genes, furthermore it measures microsatellite instability and tumor mutational burden. The molecular analysis showed no clinically relevant mutations, there was no microsatellite instability, and the total tumor mutational burden was 1.6 mutations per megabase.

At the first outpatient visit, the patient asked if the occurrence of his SDC could be somehow related to direct contact with Cr(VI) in work-related exposures in the past. A detailed occupational history revealed that the patient had been working as inspector of construction cranes and harbor cranes since 1984 up until the occurrence of his SDC disease. Initially this inspection work did not involve any use of chemicals until, in 2012, he was asked to perform inspections at three military air bases in the Netherlands over a period of 2–3 months a year. This liquid penetrant inspection was performed to detect hairline cracks in welds of military equipment. For this task, the coating was completely removed by mechanical grinding. The topcoat known as chemical agent resistant coating (CARC) contains the toxic hexamethylene diisocyanate (HDI) and is typically adhered to the metal surface with a chromic acid-containing conversion coat and primer coat containing a chromate pigment. Both are Cr(VI)-based chemical components that generate airborne particles when mechanically abrased (17). According to the patient, this procedure resulted in visible dust clouds that caused work clothes to become contaminated. No respiratory protective equipment was used and protective gloves were not always used. During breaks the worker cleaned hands but did not change clothes. When the patient blew his nose after these activities this resulted in a dusty nose secretion matching the color of the paint, indicating a clear direct exposure of the nasal mucosa to the paint grinded to dust. As of 2015, the use of the destructive method was discontinued and replaced by an alternative non-destructive inspection method.

**Figure 3:**
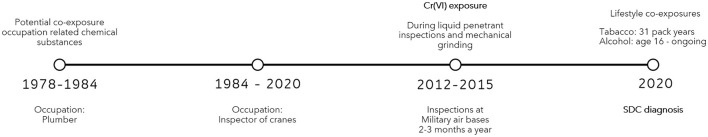
Time-line of case report 1.

Apart from Cr(VI) exposure, the patient may have come into contact with other chemical substances during a previous occupation (Figure 3). From 1978 till 1984 he worked as a plumber. Furthermore, the patient had a history of smoking and alcohol consumption. The patient started smoking shag from the age of 16 until 58, with an average of 50 gram of tobacco per week which results in a total of 31 pack years. He started to use alcoholic beverages at the age of 16 and consumes ~24 units of alcohol per week.

The patient has been treated with combined androgen deprivation therapy, because of androgen receptor expression. Previous research indicated that this therapy might alter the prognosis of patients. Without therapy patients with advanced disease had a poor overall survival of 5 months, while with androgen deprivation therapy the overall survival was 17 months (18). Therapy consists of the combination of a luteinizing hormone-releasing hormone (LHRH) agonist, goserelin subcutaneous 10.8 mg every 12 weeks, and an androgen receptor antagonist, bicalutamide tablet 50 mg once daily. Evaluation CT scans performed 3 months after start of therapy showed a partial response according to the RECIST 1.1 criteria for tumor response evaluation (19). The most recent evaluation, 9 months after initiation of treatment, showed an ongoing partial response.

### Case 2

**Figure 4:**
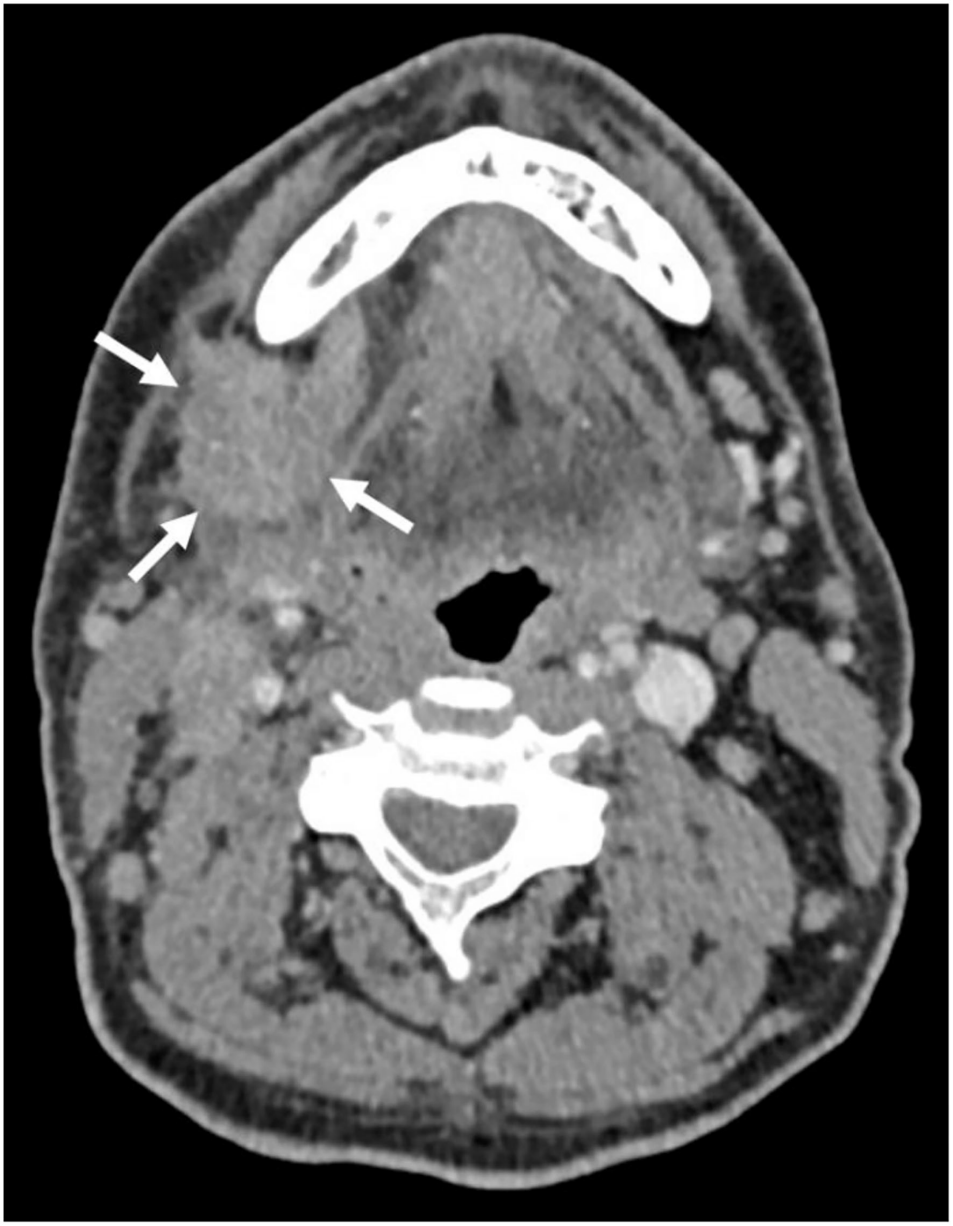
CT image of primary submandibular SDC tumor (indicated by white arrows).

**Figure 5:**
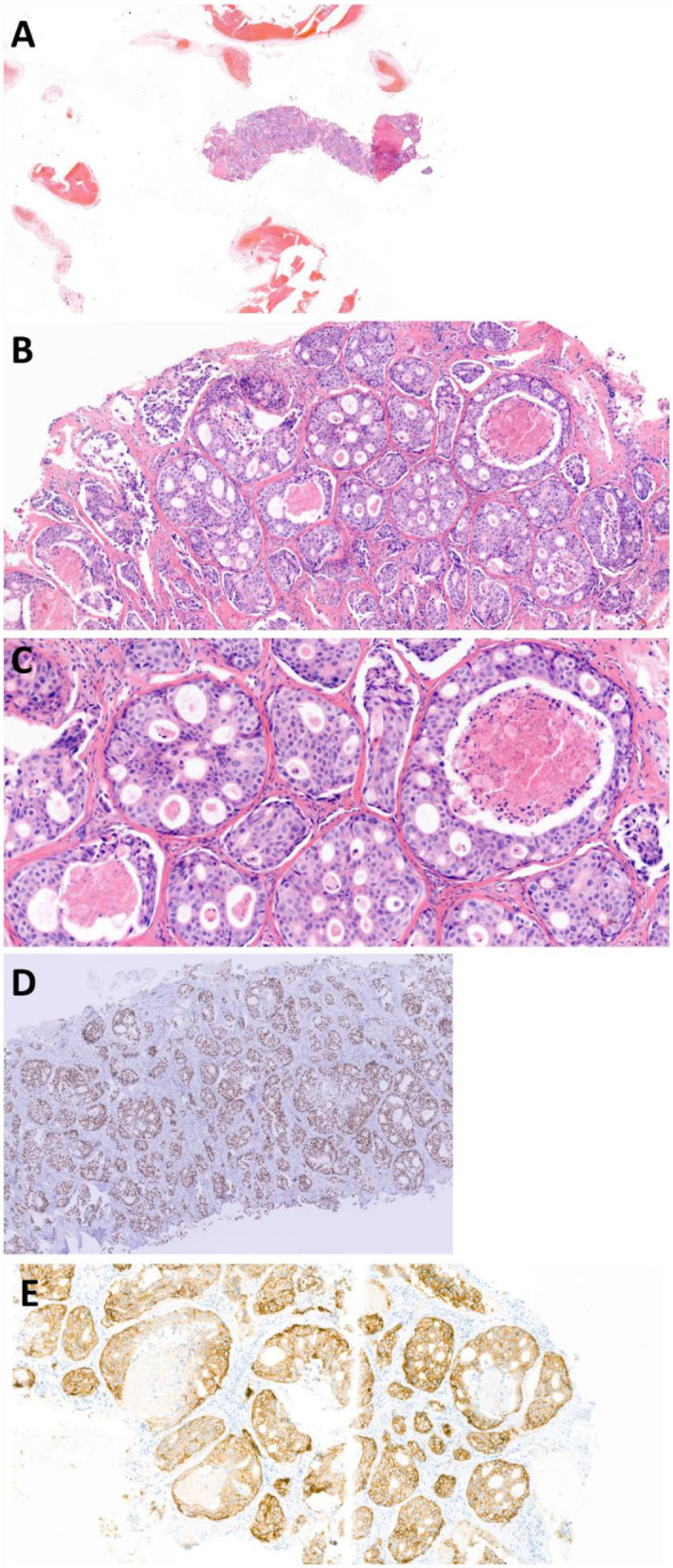
Photomicrographs of case report 2: biopsy of SDC bone metastasis in thoracic vertebra 3. 1. **(A)** H&E stain 1X, **(B)** H&E stain 10X, **(C)** H&E stain 20X, **(D)** AR stain 5X (photomicrograph from referring center), **(E)** HER2neu stain 10X, HercepTest (Dako Agilent).

A 54-year-old male was referred to a local hospital with a palpable mass in the neck at level 2 and at the right submandibular gland, suspicious for a malignant tumor. PET/CT imaging showed a primary tumor originating from the right submandibular gland (Figure 4) and also right lymphadenopathy at level I-V, suspect for lymph node metastases, and a lesion in the 3rd thoracic vertebra. Biopsy of the bone lesion revealed a SDC metastasis (T2N2bM1 disease) (Figure 5). The biopsy was largely occupied by a tumor. The tumor was made up of tubes and cribriform tubes of atypical epithelial cells with round-oval nuclei that varied in shape and size and often had a prominent nucleolus and ample amphophilic well-delimited cytoplasm. In some of the tubes necrosis was seen. Mitoses were observed. The pathology assessment concluded a bone metastasis from a salivary duct carcinoma. The patient was referred to our hospital for palliative systemic treatment. The tumor cells were positive for AR on IHC and negative for Her2neu (IHC: 2+ staining, but no amplification on FISH). Molecular analysis, using a similar assay as for case 1, presented a mutation in TP53 and CKD12, but overall, no druggable targets. Furthermore, no microsatellite instability was observed and the total tumor mutational burden was 4.7 mutations per megabase. Additionally, the assay showed many fluctuations in number of readings of different chromosomes, suggesting that there were many numerical chromosomal aberrations, which is indicative of genomic instability.

During the first outpatient visit, this patient's history also revealed an occupational exposure to Cr(VI). The patient started working as a paramedic in the army at the age of 18 and has worked there for a total 32 years. In addition to his nursing duties, he was involved in maintenance of 4 × 4 military trucks type YA-314 and YA-328 (~2 h/day). In the years 1987–1992 old paint was removed by manual abrasion using steel brush and paint scraper. New primer and topcoat were applied by spray and brush painting. In the period 1992–2017 the work continued but painting was mainly done by spot painting. Both tasks were performed indoor in an unventilated large workshop without the use of a spray booth or local exhaust ventilation for ~4 h/month. During spray painting this resulted in substantial exposures to volatile organic compounds from the thinner used. Personal protective equipment such as respirators or gloves were not used and not available. The paint was very sticky and could remain on the hands even when washing hands with water and soap prior to short breaks (for smoking) and longer breaks for a meal in the recreation room. Contaminated work garment was not changed prior to breaks. Exposure to organic solvents from paint and thinner was indicated by a bad mouth taste. Apart from occasional headaches no acute signs of neurotoxicity were reported on the day of exposure or the day after.

**Figure 6:**
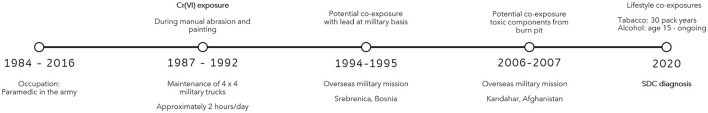
Time-line of case report 2.

In addition to Cr(VI) exposure, the patient may have come into contact with other chemical substances (Figure 6). The patient was sent overseas for military missions for 3 months at the mission headquarters in Srebrenica, Bosnia that was set up on a former industrial site as part of UNPROFOR in 1994–1995 (20). There were remains of a lead battery factory that was partly destroyed in the conflict. The site was heavily contaminated with lead-containing dust. Lead exposure was confirmed by blood analyses in some of the military staff but did not results in health complaints (21). The patients himself also reported no symptoms of lead exposure. Additionally, he worked as paramedic in Kandahar, Afghanistan (ISAF) in 2006–2007 (22). There, depending on the wind direction the smoke from a nearby burn pit would blow through the compound causing complaints of very bad smell indicating exposure to smoke fumes with unidentified toxic components (23). Lifestyle exposures included; consumption of alcoholic beverages, from the age of 15, ~5 units of alcohol per week, and smoking with an average of 20 cigarettes per week during 30 years (30 pack years).

The patient started androgen deprivation therapy, in the form of bicalutamide monotherapy (tablet 150 mg once daily) because of androgen receptor positivity. Unfortunately, first treatment evaluation, 3 months after start, showed progressive disease (RECIST v1.1). The patient was subsequently referred for palliative radiotherapy and start of palliative chemotherapy.

## Discussion and Conclusion

Here we presented two SDC patients both with a history of Cr(VI) exposure related to mechanical abrasion of chromate containing metal coating in maintenance of military equipment. This triggers the question of a potential relation between the occurrence of SDC and Cr(VI) exposures. To address this question we will first describe known risk factors for SDC and then we will focus on the toxic effects of Cr(VI) and their potential relation to the occurrence of SDC and finally on the prevention of Cr(VI)-exposure.

Little is known about risk factors for SDC specifically, but several studies have investigated potential risk factors for salivary gland cancer. While smoking and alcohol are a clear risk factor for other types of head and neck cancer, especially squamous cell carcinoma (24), most studies showed no or limited association between smoking tobacco or alcohol consumption and salivary gland cancer (25–30). Previously addressed risk factors for developing salivary gland cancer include: prior radiation to the head or neck during radiotherapy or during dental or cervicofacial radiological examinations, occupational exposure to radioactive materials and nickel compounds/alloys, ambient air pollution from waste gas emissions and certain occupations, like plumber, sheet-metal worker, and building painter (5, 30, 31). Yet, Cr(VI) exposure is not mentioned specifically in these reports. Our literature search on Cr(VI) exposure and SDC through PubMed did not yield any relevant articles, also indicating that a possible association has not yet been discussed in literature. Expanding the search to salivary gland cancer resulted in one relevant article (32). In South Korea the incidence of respiratory tract cancers was studied as a function of the distance to Portland cement plants. These plants are known to result in elevated combined emissions of chromium, crystalline silica and polycyclic aromatic hydrocarbons over long distances. The incidence of salivary gland cancer near the cement plants was ~3 times higher in females (standardized incidence ratio: 3.03, 95%CI: 0.98–7.07), using the national incidence in the South-Korean female population as comparator. Evidence for exposure of salivary glands specifically to Cr(VI) was not found but is considered plausible regarding the inhalable size of the cement dust particles.

Regarding the carcinogenic effects of Cr(VI) exposure; Cr(VI) can cause oxidative DNA damage, which in turn could increase the risk of neoplastic formation, especially in the case of reoccurring exposure (33, 34). Cr(VI) containing chromate ions are thought to enter cells by a shunt that is normally used for uptake of sulfate and phosphate and may reach the nucleus (35). When Cr(VI) is reduced reactive oxygen is generated, leading to oxidative and other types of DNA damage confirmed in cell cultures (36). It is still unclear to what extent genotoxicity by itself or combined with epigenetic/inflammatory processes leads to DNA instability, impaired apoptosis and inhibited normal DNA-repair which may all explain tumor induction in animal experiments (37). Interestingly, In both patients molecular analysis has been performed. We did not find remarkable anomalies in case report 1 but in the case report 2, a mutation in TP53 was observed and the assay indicated genomic instability. Mutations in TP53 are common in SDC, present in 53–68% of the tumors (7, 38). The genomic instability can be the result of many possible causes, including TP53 mutations, but has also been previously reported as a result of prior Cr(VI) exposure (39, 40).

In addition to the rarity of SDC itself, in both patients the primary tumor originated at an unusual location. SDC mostly arises from the parotid gland (72% of the cases). In the first patient the SDC originated in the sinonasal tract, which is extremely rare, only four other reports of primary SDC originating in the sinonasal tract have been reported (41). In the second patient the SDC occurred in the submandibular gland, ~15% of SDC occur in this salivary gland (42).

The International Agency for Research on Cancer (IARC) classified Cr(VI) as a group 1 human carcinogen based on evidence from population-based studies and experimental animal studies both suggesting an increased risk of lung cancer following occupational exposure to Cr(VI) (13).

In our patient from case report 1 however, cancer arose in a different part of the airway system, namely as mentioned before in the sinonasal tract. A preclinical study which investigated the effect of inhalation of chromium by exposing mice for 12 months to chromic acid mist several mice developed nasal tumors (papillomas) (43). As for sinonasal cancer, the IARC assessed epidemiological evidence for Cr(VI) exposure as the cause for sinonasal cancer and stated that an association was suggestive, however inconclusive (13). Since then, more research regarding this topic has been published. A systematic review and meta-analysis estimated the relative risk of sinonasal cancer to be 18.0 (95%CI: 14.6–22.3) in workers who work with chromium and nickel (44). Another report on Cr(VI) exposure and sinonasal cancer described a maximum relative risk for developing sinonasal cancer among chromate production workers of 15.4 (45). In a recently updated narrative literature review prepared by the Dutch Institute for Public Health and the Environment (RIVM) the available evidence from human observational combined with experimental animal evidence was evaluated as sufficient to conclude that Cr(VI) exposure can be considered as a risk factor for nose and sinonasal cancer (46).

In the second patient the SDC arose from the submandibular gland. As mentioned before, little has been described in the literature about the association between salivary gland cancer and Cr(VI) exposure. For the submandibular gland tissue there some supporting evidence but not a clear and direct link with occupational Cr(VI) exposure. Oral exposure to Cr(VI) is a well-known in oral carcinogenicity (47). Cancer of the oral cavity has been observed in rats who received Cr(VI) in drinking water (48). In workers in metal industry with primarily inhalation exposure dental injury was observed, more specifically a higher incidence of dental caries and mastication deficiency was observed in 100 patients with occupational exposure to chromates in an industrial setting (49).

Apart from Cr(VI) exposure, co-exposure to other carcinogenic substances should always be considered as a contributing factor for the occurrence of cancer. When we considered other potential exposures in our patients, we first evaluated other chemical substances arising from the maintenance of military equipment. Apart from the chromate in the CARC (topcoat of the metal surface), HDI is considered the most toxic ingredient (50). HDI is a known respiratory sensitizer, but has not been classified as a carcinogen and is therefore unlikely to have contributed to the occurrence of SDC (51). Furthermore, our first patient was exposed to fumes from soldering when he worked as a plumber. In these fumes human carcinogens such as nickel oxide and chromates are not often encountered, but aldehydes are (52, 53). Specifically, formaldehyde is a known risk factor of nasal and sinonasal cancer and might have contributed to the occurrence of SDC in this patient (54). Finally, both patients had a history of tobacco and alcohol consumption. The medical file indicated no history of radiation exposure for diagnostic or therapeutic purposes prior to the SDC diagnostic work-up. However, a contribution of this to the occurrence of SDC is considered less likely, given the lack of a clear association with salivary gland cancer as mentioned above.

Because of the deleterious effects of Cr(VI) it is important to minimize or prevent exposure of employees as much as possible. Occupational exposure to Cr(VI) is currently regulated in EU with a binding Occupational Exposure Limit (OEL) expressed as 8-h time-weighted average (8-h TWA) of 10 μg/m^3^ with the intention to further limit this OEL to 5 μg/m^3^. The Netherlands and France have currently worldwide the strictest 8-h TWA of 1 μg/m^3^ (55, 56). These OELs have been derived based on human lung cancer risk in workers for which there is no evidence for a threshold. So, an exposure below the OEL does not mean that there is no residual cancer risk. In the Netherlands the OEL was derived from a residual accepted risk of 1 × 10^−4^ per exposure year (corresponding to a risk 4 × 10^−3^ for a working life exposure of 40 years) (57). The first priority is to comply with the legally binding OEL. When this is achieved further reductions of exposure are mandatory in line with international technical state-of-the-art. For this the occupational hygiene strategy is applied: if the Cr(VI) can not be eliminated or substituted with an alternative substance with a lower health risk, risk management measures (RMM) are implemented to minimize exposure by segregation of the workers from the source (e.g., by containment of the process or moving the operator at a safe distance from the source), installment of local air ventilation and additional room ventilation, changing the lay-out of the workroom and/or organizing the work differently, and as a last resort, introduce personal protective equipment such as providing respirators with high-efficient particle filters.

Recently, it was suggested that in addition to direct inhalation exposure also dermal exposure was associated with uptake of Cr(VI) (58). So indirect exposure caused by secondary contamination should also be avoided to prevent exposure by good personal hygiene, e.g., by providing protective gloves and overalls with long sleeves, allowing no consumption of food/beverages or smoking at the workplace and providing standard water sanitation and hygiene facilities and additional facilities for full body decontamination for emergencies. For proper implementation of these measures an occupational hygienist and occupational physician should be involved to perform exposure and health surveillance.

In conclusion, our report highlighted two patients with SDC which have both been exposed to Cr(VI). Mechanical abrasion of painted metals of military equipment as part of corrosion control is a known source of inhalation exposure exceeding current standards for protection of worker's health (17). A causal relation between Cr(VI) exposure and SDC is difficult to prove both on individual as well as population basis given the extreme rarity of SDC. Nevertheless, we consider it plausible that Cr(VI) containing chromates might have contributed to the occurrence of the disease as the common factor in both cases as exposures occurred in a poorly ventilated working environment (no local exhaust ventilation) and the work was conducted without appropriate respiratory protective equipment. For case 1 we cannot rule out a contribution from working as a plumber which may also have resulted in exposure to formaldehyde. With this report we aim to increase the awareness of occupational Cr(VI) exposure and occurrence of SDC. Furthermore, we want to emphasize the importance of primary prevention in order to minimize exposure to chemical carcinogens.

## Data Availability Statement

The original contributions presented in the study are included in the article/supplementary material, further inquiries can be directed to the corresponding author.

## Ethics Statement

Ethical review and approval was not required for the study on human participants in accordance with the local legislation and institutional requirements. The patients/participants provided their written informed consent to participate in this study. Both patients gave written informed consent for use of their medical history and radiological images in this paper.

## Author Contributions

All authors contributed to writing this manuscript, read, and approved the final manuscript.

## Conflict of Interest

The authors declare that the research was conducted in the absence of any commercial or financial relationships that could be construed as a potential conflict of interest.

## Publisher's Note

All claims expressed in this article are solely those of the authors and do not necessarily represent those of their affiliated organizations, or those of the publisher, the editors and the reviewers. Any product that may be evaluated in this article, or claim that may be made by its manufacturer, is not guaranteed or endorsed by the publisher.

## Abbreviations

AR: Androgen receptor
Cr(VI): Chromium VI
CARC: Chemical agent resistant coating
H&E: Hematoxylin and eosin stain
HDI: Hexamethylene diisocyanate
HER2neu: Human epidermal growth factor receptor 2
IARC: International Agency for Research on Cancer
ISAF: International Security Assistance Force
LHRH: Luteinizing hormone-releasing hormone
OEL: occupational exposure level
RMM: risk management measures
SDC: Salivary duct carcinoma
UNPROFOR: United Nations Protection Force.
